# Supplementary material for: Assessment of global health risk of antibiotic resistance genes
Source: Nat Commun. 2022 Mar 23;13:1553. doi: 10.1038/s41467-022-29283-8 (PMC8943045; doi:10.1038/s41467-022-29283-8)
Supplement: Supplementary file 1 — Supplementary information [file 41467_2022_29283_MOESM1_ESM.pdf]

Supplementary information for

Assessment of global health risk of antibiotic resistance genes

Zhenyan Zhang[1,†], Qi Zhang[1,†], Tingzhang Wang[2,†], Nuohan Xu[1],Tao Lu[1], Wenjie Hong[2], Josep Penuelas[3,4], Michael Gillings[5], Meixia Wang[2], Wenwen Gao[2], Haifeng Qian[1,*]

1. College of Environment, Zhejiang University of Technology, Hangzhou 310032, P. R. of China.
2. Key laboratory of microbial technology and bioinformatics of Zhejiang Province, Hangzhou 310012, P. R. of China.
3. CSIC, Global Ecology Unit CREAF- CSIC-UAB, Bellaterra, Barcelona 08193, Catalonia, Spain.
4. CREAF, Campus Universitat Autònoma de Barcelona, Cerdanyola del Vallès, Barcelona 08193, Catalonia, Spain.
5. ARC Centre of Excellence in Synthetic Biology, Faculty of Science and Engineering, Macquarie University, NSW 2109, Australia.

[†]Zhenyan Zhang, Qi Zhang and Tingzhang Wang contributed equally to this work.

[*]Correspondence to: Haifeng Qian (hfqian@zjut.edu.cn)

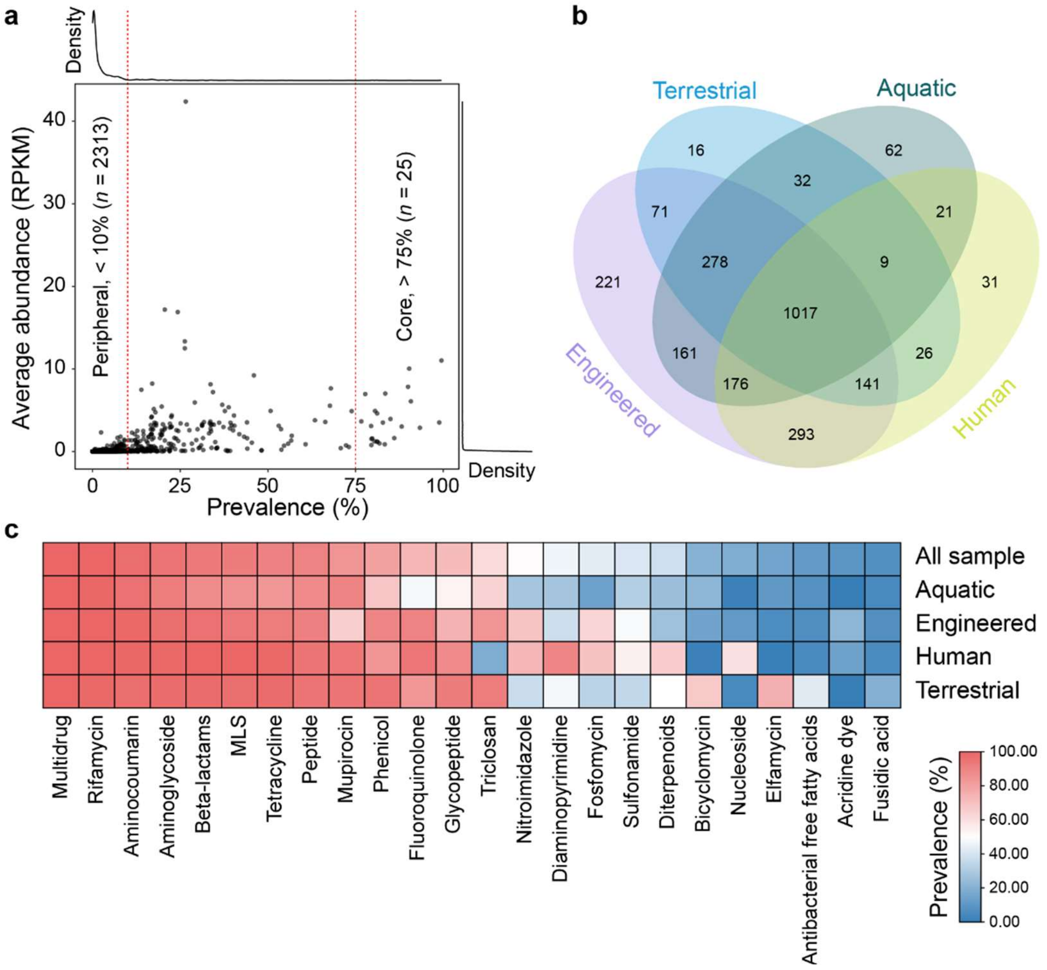

Supplementary Fig. 1 Distribution of ARGs across samples and habitats. a, Average abundance and prevalence of ARGs in all samples (*n* = 4572 samples). The upper and right panels show the distribution of prevalence and abundance, respectively. Most ARGs (2313/2561) are detected in less than 10% of samples**. b,** Shared and specific ARGs in diverse habitats. Most ARGs are found in diverse habitats. **c,** Prevalence of ARGs with resistance to particular drug classes in all samples and specific habitats. Several ARG classes have distinct distribution patterns.

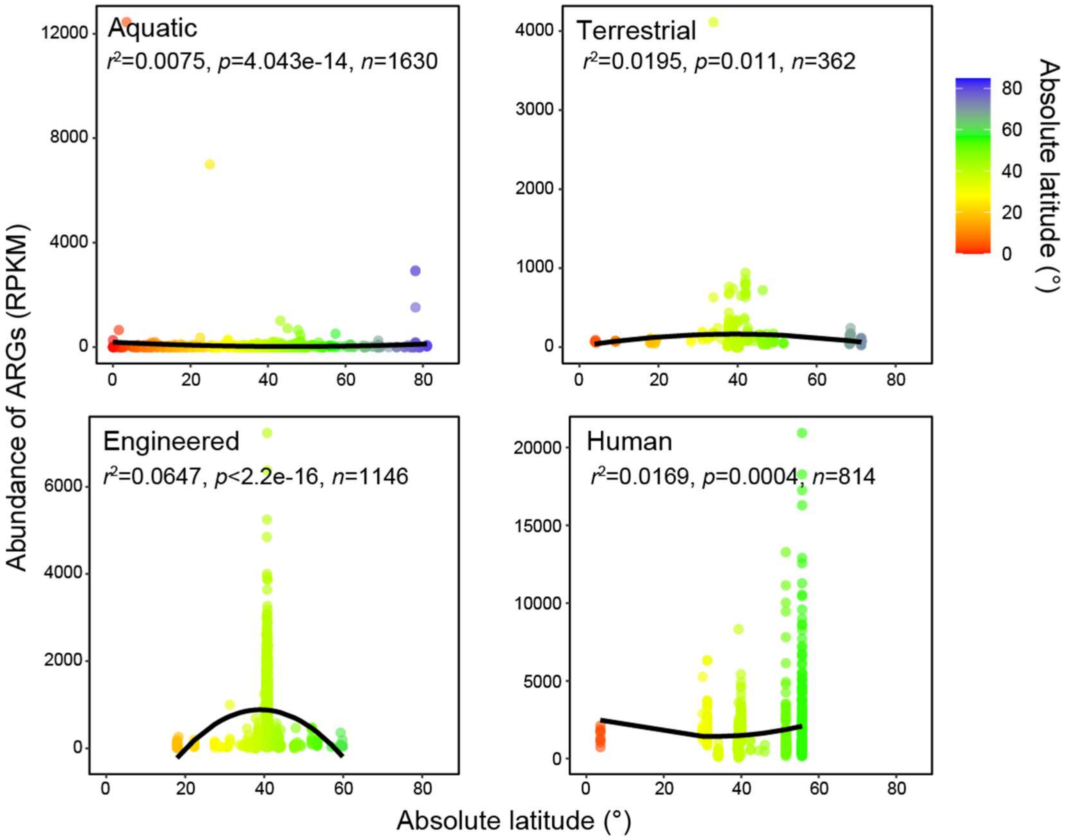

Supplementary Fig. 2 Latitude influences the abundance of ARGs in four main habitats. Black solid lines are the second order polynomial regression lines. The r^2 represents the R-squared of regression. The *p* value represents statistical significance, evaluated by F-statistic (one-sided). And the *n* represents the number of biologically independent samples in each habitat.

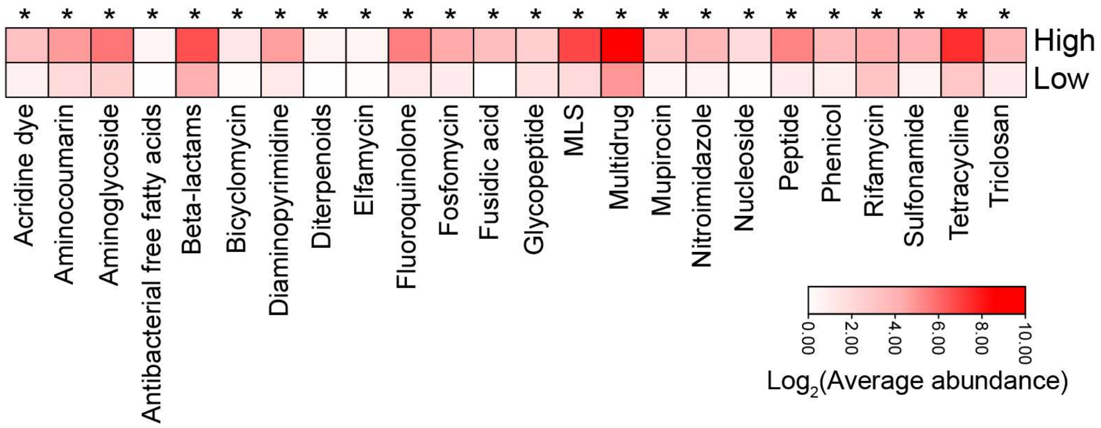

Supplementary Fig. 3 High-intensity human activities significantly promote the abundance of ARGs. *n* = 1643 and 2309 biologically independent samples for Low- and High-group, respectively. * represents the statistical significance (adjust $p < 0.05$; two-tailed Welch's t-test). All adjust *p* values are provided in the Source Data 5.

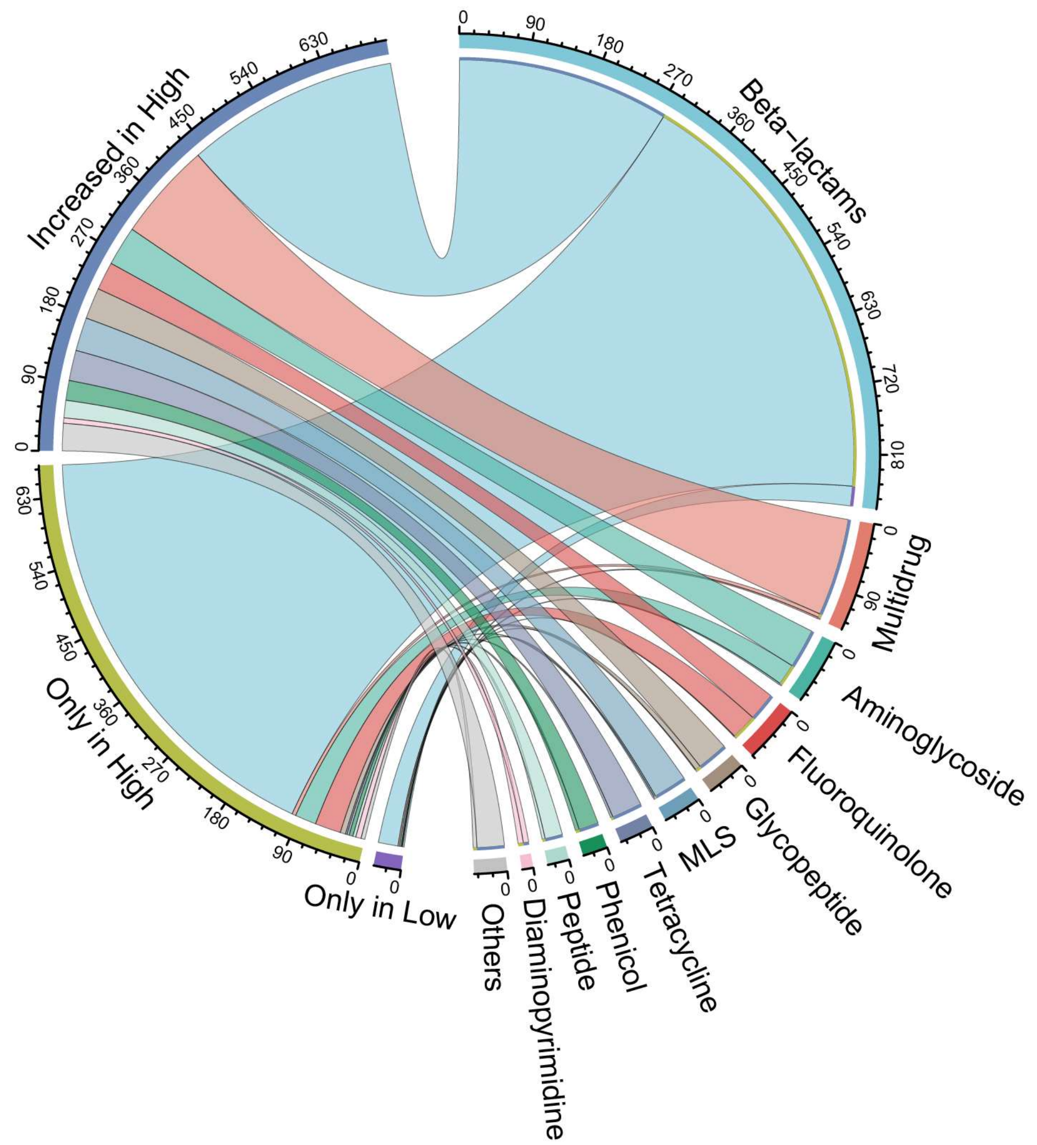

Supplementary Fig. 4 Drug classes of ARGs shared or specific to the areas with low or high human activities. The number of ARGs in each group and drug class is shown. ARGs increase in high-intensity human activity areas compared with the low one.

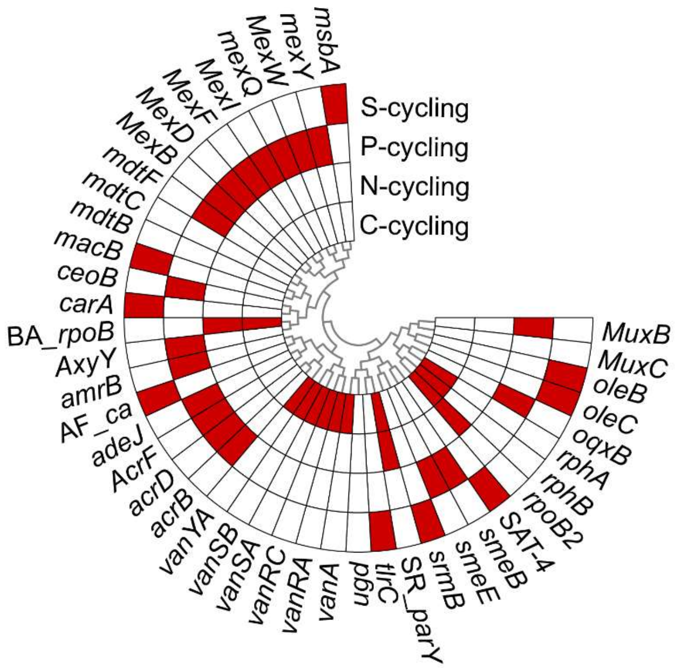

Supplementary Fig. 5 ARGs carry out biological functions besides antibiotic resistance. Only shows the ARGs carry out biological functions besides antibiotic resistance. BA_*rpoB*: *Bifidobacterium adolescentis rpoB* conferring resistance to rifampicin; AF_*ca*: *Agrobacterium fabrum* chloramphenicol acetyltransferase; SR_*parY*: *Streptomyces rishiriensis* parY mutant conferring resistance to aminocoumarin.

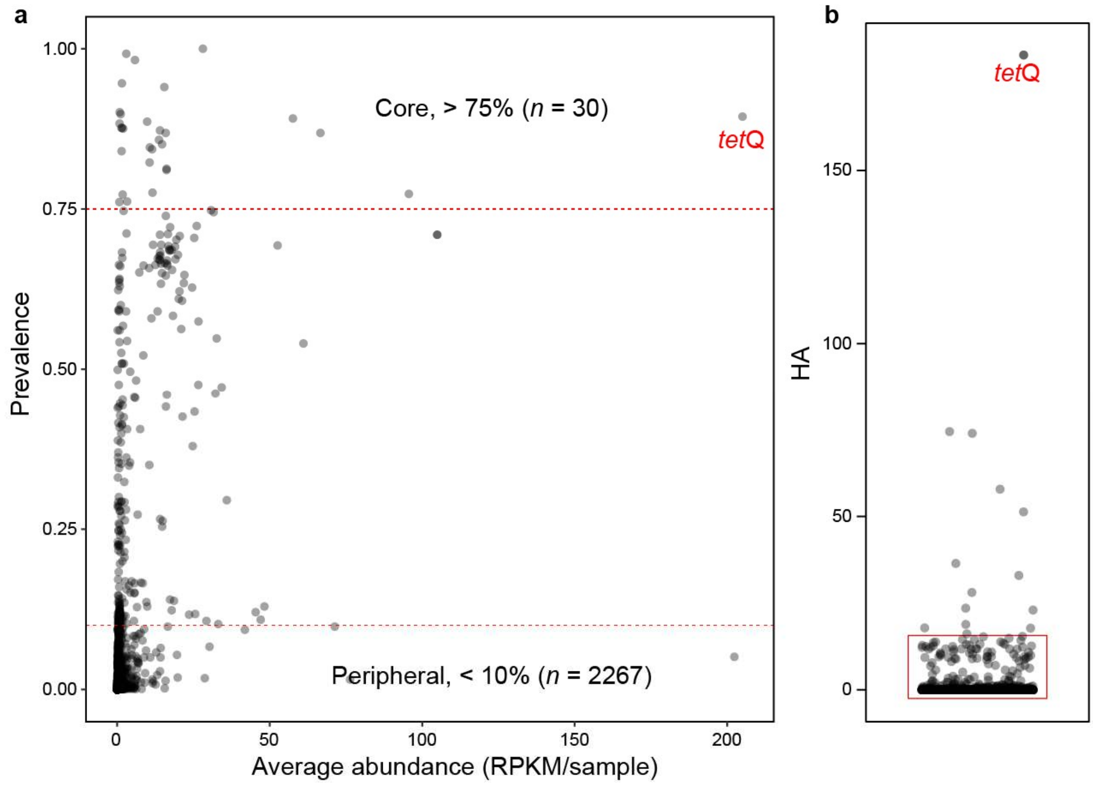

Supplementary Fig. 6 Human accessibility of ARGs. a, Average abundance and prevalence of ARGs in human-associated habitats. **b,** Human accessibility calculated by the abundance and prevalence. *tet*Q, conferring resistance to the tetracycline antibiotics, is the most prevalent ARG in human-associated habitats and results in the highest human accessibility. HA: human accessibility.

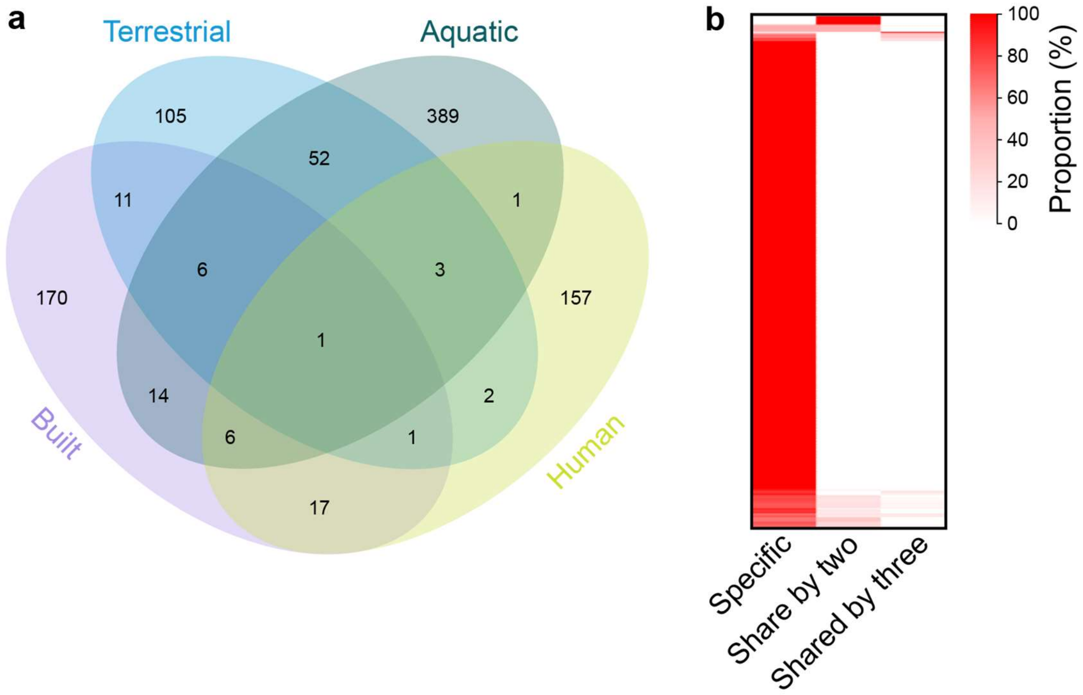

Supplementary Fig. 7 Distribution of host of ARGs in diverse habitats. a, Venn plot of shared and specific hosts which carried ARGs in diverse habitats. **b,** Proportion of hosts specific or shared by multiple habitats for each ARG. The hosts of ARG are almost completely different in different habitats.

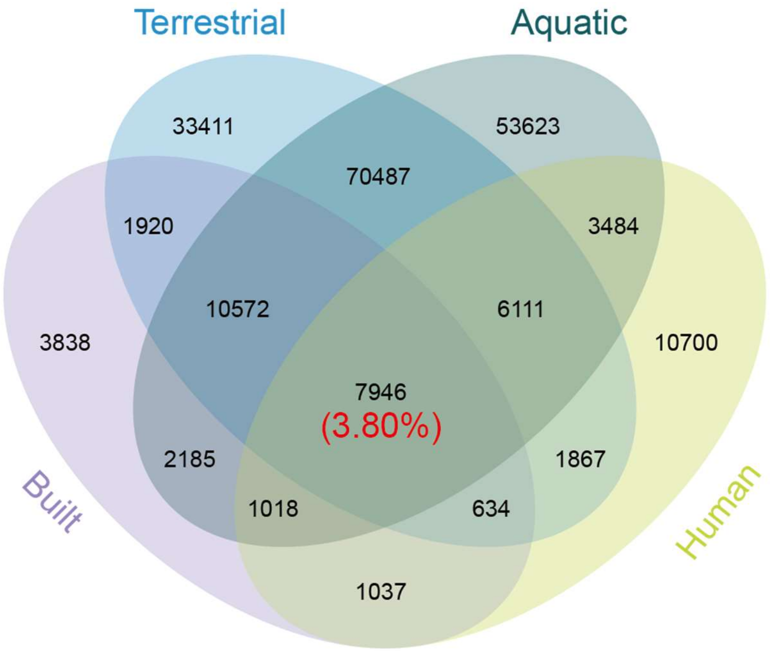

Supplementary Fig. 8 Distribution of MGEs in diverse habitats. Venn plot of shared and specific MGE in diverse habitats. There are only 3.80% MGEs shared in different habitats.

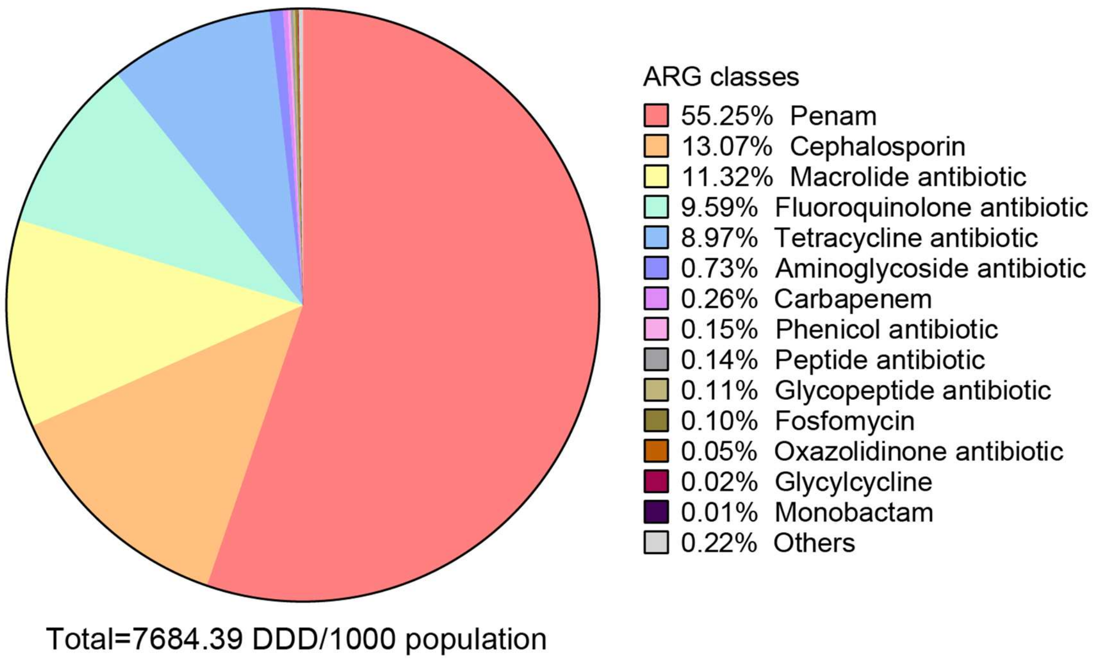

Supplementary Fig. 9 Global consumption of antibiotics. Data were collected from (https://resistancemap.cddep.org/AntibioticUse.php) in August 2021. DDD: defined daily doses. Penam (55.25%) and cephalosporin (13.07%), two beta-lactam antibiotics, occupied the largest antibiotic market.

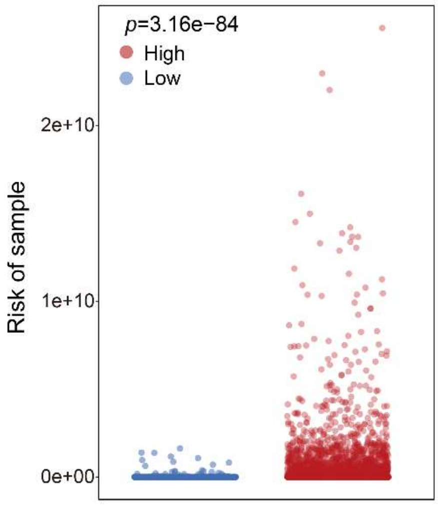

Supplementary Fig. 10 Risk index of samples in low- or high-intensity human activities areas. High-intensity human activities increase the health risk of ARGs for humans. n = 1643 and 2309 biologically independent samples for Low- and High-group, respectively. The p value represents the statistical significance by two-tailed Welch's t-test.

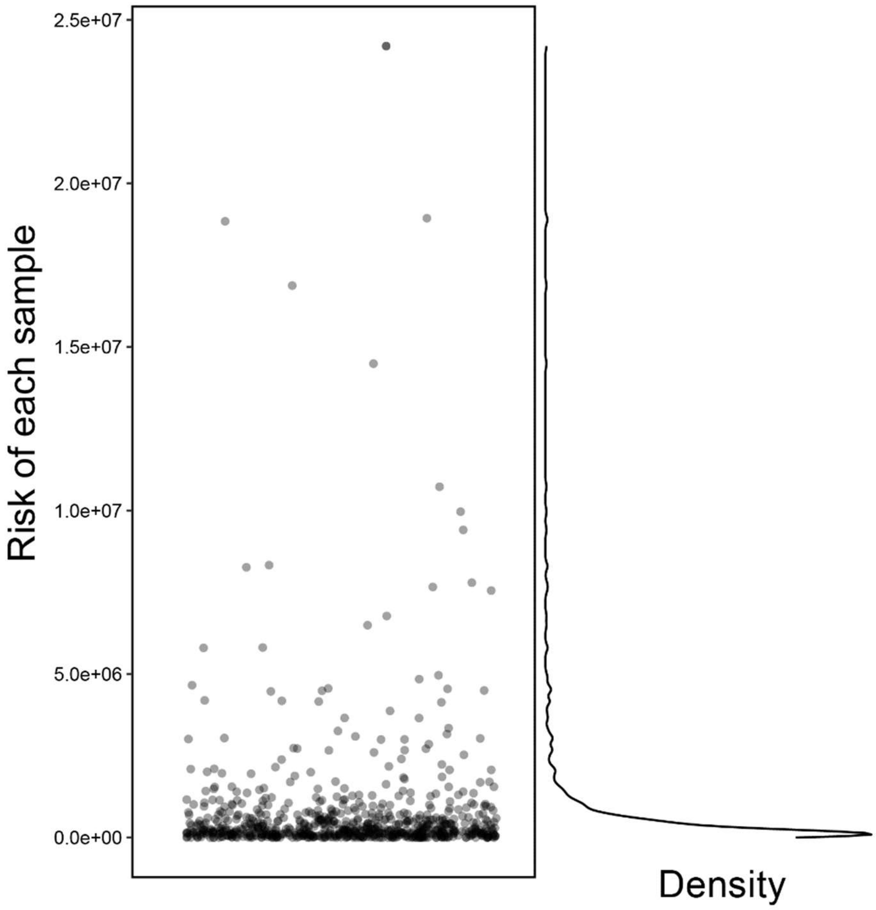

Supplementary Fig. 11 The risk of 712 marine samples used for machine learning.

The distribution of the risks for marine samples are uneven.

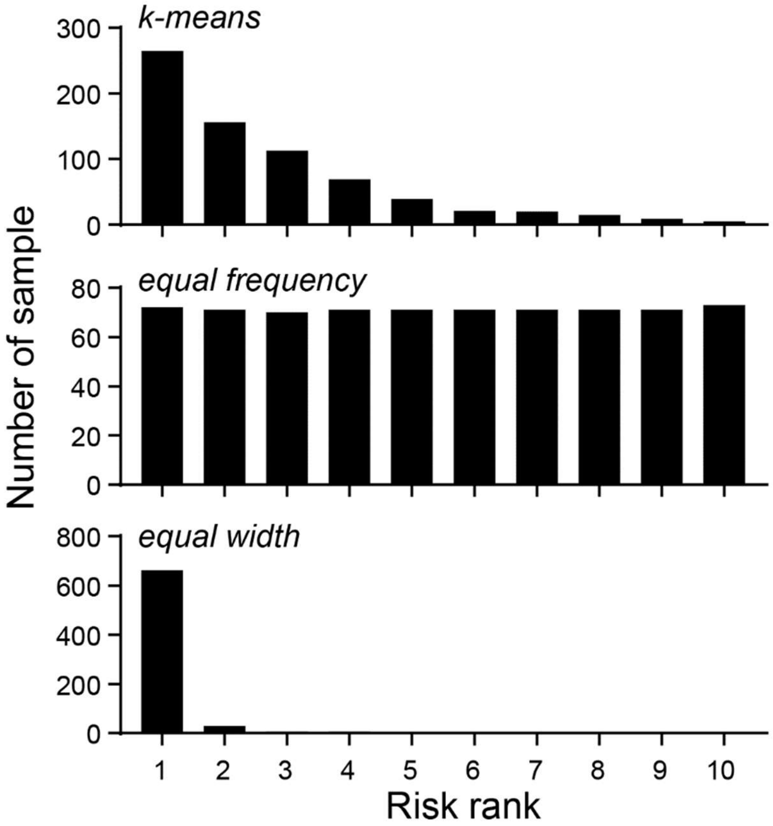

Supplementary Fig. 12 The number of samples in each risk rank after discretization by three methods. Nearly all the samples are classified as rank 1 after discretization by equal width.

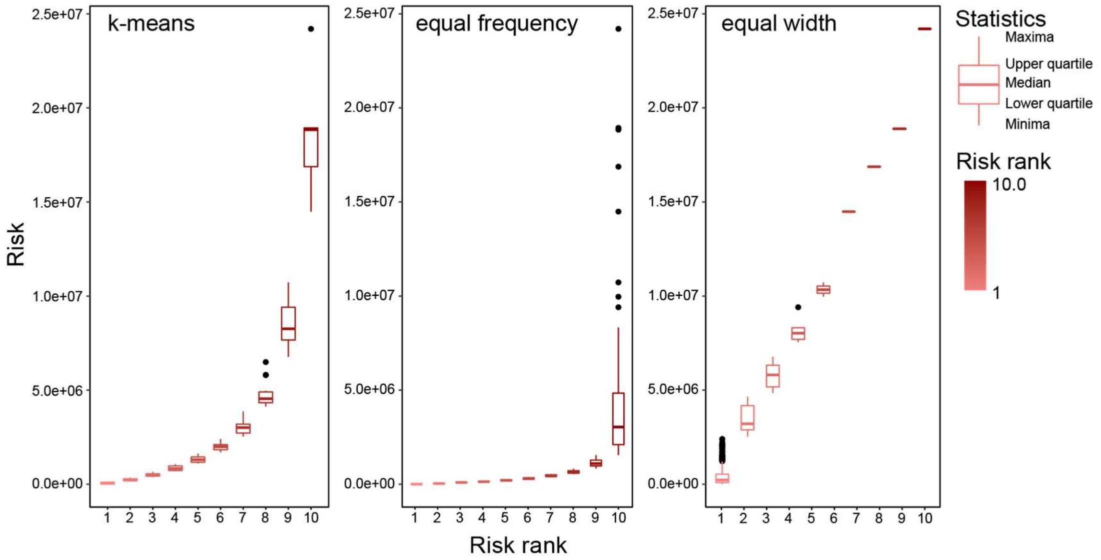

Supplementary Fig. 13 The distribution of risks in samples (*n* = 712 biologically independent samples) from each rank after discretization by three methods. The black points represent the outlier of box. Samples after discretization by equal frequency show low discriminability in ranks 1 to 5.

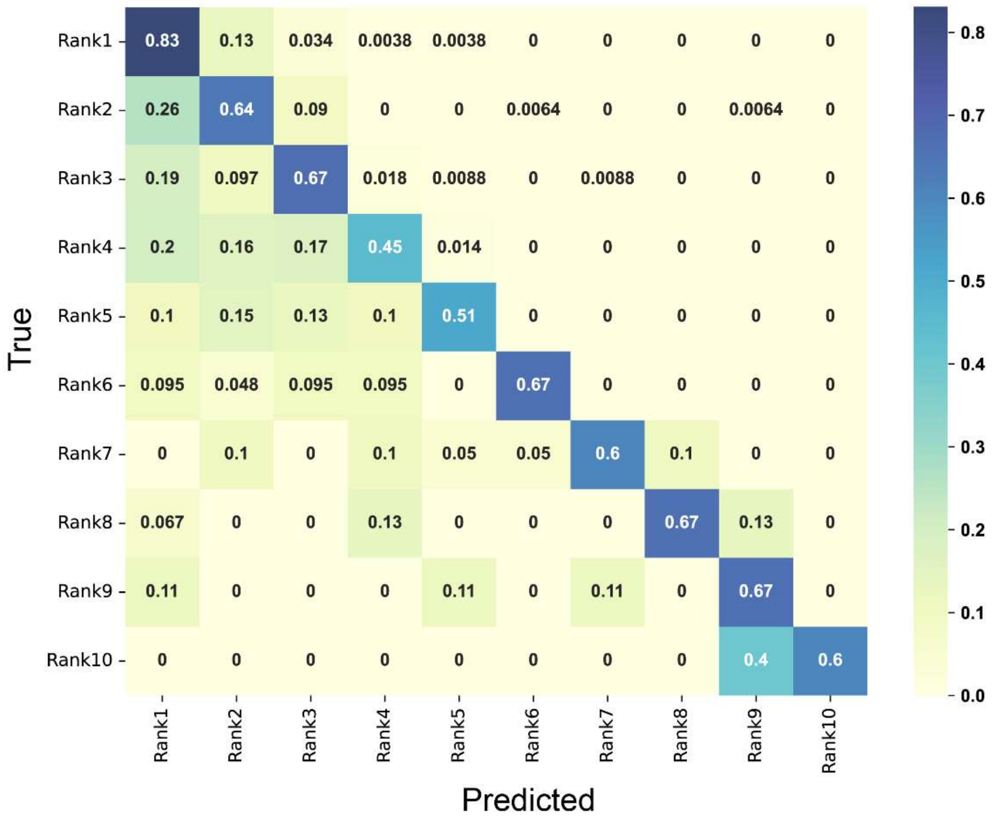

Supplementary Fig. 14 The confusion matrix elements denote the number of each predicted rank in machine learning with random forest algorithm. 90% of the data were randomly selected for the random forest algorithm for the training of softness classification and recognition, and the remaining 10% data were used as the test data. The number of the true prediction results by machine learning is recorded on the horizontal line and expressed as the frequency.
