# Supplementary material for: Assessment of global health risk of antibiotic resistance genes
Source: Nat Commun. 2022 Mar 23;13:1553. doi: 10.1038/s41467-022-29283-8 (PMC8943045; doi:10.1038/s41467-022-29283-8)
Supplement: Supplementary file 3 — Description of Additional Supplementary Information [file 41467_2022_29283_MOESM3_ESM.docx]

**Description of Additional Supplementary Files**

**File Name:** Supplementary Data 1

**Description:** Metadata of 4572 metagenomic samples used in this study.

**File Name:** Supplementary Data 2

**Description:** ARG annotation and abundance calculation for each sample.

**File Name:** Supplementary Data 3

**Description:** Results of two-tailed Welch’s t-test of all 1846 shared ARGs.

**File Name:** Supplementary Data 4

**Description:** Human accessibility of 2561 ARGs evaluated in this study.

**File Name:** Supplementary Data 5

**Description:** Hosts of ARG identified by MAGs with strict quality criteria.

**File Name:** Supplementary Data 6

**Description:** Metadata and ARGs annotation of 27013 completed genomes used in this study.

**File Name:** Supplementary Data 7

**Description:** Links between ARGs and MGEs in completed genomes.

**File Name:** Supplementary Data 8

**Description:** Human pathogenicity of ARGs calculated by the proportion of pathogenic hosts.

**File Name:** Supplementary Data 9

**Description:** Clinical availability of ARGs calculated by the total use of antibiotics for each class of ARG.

**File Name:** Supplementary Data 10

**Description:** Quantitative risk of 2561 ARGs detected in metagenomic analysis and their ranks.

**File Name:** Supplementary Data 11

**Description:** Proportion of ARGs in each rank detected in the hospital pathogenic MAGs.

**File Name:** Supplementary Data 12

**Description:** Information of 712 marine samples.

**File Name:** Source Data

**Description:** The raw data underlying figures.
